# Genetic variants of *NOXA* and *MCL1* modify the risk of HPV16-associated squamous cell carcinoma of the head and neck

**DOI:** 10.1186/1471-2407-12-159

**Published:** 2012-05-01

**Authors:** Ziyuan Zhou, Erich M Sturgis, Zhensheng Liu, Li-E Wang, Qingyi Wei, Guojun Li

**Affiliations:** 1 Departments of Epidemiology, The University of Texas MD Anderson Cancer Center, Houston, Texas, USA; 2 Head and Neck Surgery, The University of Texas MD Anderson Cancer Center, Houston, Texas, USA; 3 Department of Head and Neck Surgery, Unit 1445, The University of Texas MD Anderson Cancer Center, 1515 Holcombe Boulevard, Houston, TX 77030, USA

**Keywords:** *NOXA*, *MCL1*, HPV16, Genetic susceptibility, Squamous cell carcinoma of the head and neck

## Abstract

**Abstracts:**

**Background**

The cooperation between phorbol 12-myristate 13-acetate induced protein 1 (NOXA) and myeloid cell leukemia 1 (MCL1) is critical in the intrinsic apoptotic pathway. Human papillomavirus 16 (HPV16), by inducing p53 and pRb-E2F degradation, may play an essential role in development of squamous cell carcinoma of the head and neck (SCCHN) through NOXA-MCL1 axis-mediated apoptosis. Therefore, genetic variants of *NOXA* and *MCL1* may modify the SCCHN risk associated with HPV16 seropositivity.

**Methods**

HPV16 serology was obtained by immunoadsorption assay. Four functional SNPs in the promoter of *NOXA* (rs9957673, rs4558496) and *MCL1* (rs9803935, rs3738485) were genotyped for 380 cases and 335 frequency-matched cancer-free controls of non-Hispanic whites.

**Results**

Associations between the four polymorphisms and SCCHN risk were not significant, while we observed a significantly joint effect on SCCHN risk between the polymorphisms and HPV16 seropositivity. Notably, this effect modification was particularly pronounced for oropharyngeal cancer in subgroups including never smokers, never drinkers and younger subjects.

**Conclusions**

Our results suggested that polymorphisms of *NOXA* and *MCL1* may modify the risk of HPV16-associated oropharyngeal cancer. The further identification of population subgroups at higher risk provides evidence that HPV-targeting treatment may help benefit SCCHN. However, larger studies are needed to validate our findings.

## Background

Apoptosis has been implicated in the development of various human cancers, including squamous cell carcinoma of the head and neck (SCCHN). Growing evidence suggests that suboptimal apoptotic capacity leads to failure in responding to unfavorable stimuli and eliminating potentially neoplastic clones, which may cause accumulation of cells with cancer-prone mutations and therefore result in cancer susceptibility [1]. The Bcl-2 family proteins, as well as cooperation among their anti- and pro-apoptotic subgroup members, control the balance between death (apoptosis) and survival of cells, and consequently may alter the risk of cancers [2-5]. As one of the p53 downstream targets, the pro-apoptotic Bcl-2 subfamily protein NOXA (Latin for damage; HUGO designation is phorbol 12-myristate 13-acetate induced protein 1, PMAIP1) is recognized as a crucial switch that connects apoptotic signals with the anti-apoptotic subfamily proteins. Indeed, the NOXA can exclusively bind to the anti-apoptotic subfamily protein MCL1 (myeloid cell leukemia 1) and consequently initiate apoptosis by both sequestering MCL1 and promoting MCL1 degradation [4,6,7]. Evidence from in vitro and in vivo studies indicates that NOXA deficiency or MCL1 over-expression may protect cells from apoptosis in a p53- dependent or independent manner, whereas NOXA accumulation and MCL1 inhibition/degradation may help restore sensitivity to apoptosis [7-9]. The role of NOXA-MCL1 axis-mediated apoptosis in cancer risk is not fully understood. High levels of MCL1 were found in solid human tumors and cell lines derived from human leukemia or lymphomas [10]; furthermore, studies in vivo showed that knockdown of *NOXA* or co-expression of *MCL1* and oncogene *MYC* can significantly accelerate the development of tumors such as lymphoma or leukemia [10-12]. This implies that *NOXA* and *MCL1* may be extensively involved in the tumorigenesis of different origins. However, whether *NOXA* and *MCL1* play a role in the etiology of head and neck cancer remains unclear.

As the eighth most common cancer worldwide, SCCHN accounts for nearly 3% of all incident malignancies in the U.S. with approximately 24,000 cases of oral cavity cancer, 13,000 of oropharyngeal cancer, and 12,000 of laryngeal cancer [13]. Studies have demonstrated that SCCHN is a heterogeneous group of distinct subtypes with distinct risk factors. In addition to the most well defined risk factors, tobacco smoking and alcohol drinking, the infection of human papillomavirus (HPV), 90% of which is the oncogenic type 16, is recently reported to be detectable in approximately one fourth to one third of SCCHN [14,15], and recognized as one of the most potentially contributive risk factors for SCCHN arising at the oropharyngeal site [16-22]. The major HPV oncoproteins E6 and E7 cooperate in HPV-related carcinogenesis. By E6-induced p53 degradation and E7-induced pRb-E2F degradation, HPV infection may possibly cause deregulation of p53-pathway and pRb-E2F-pathway, respectively, consequently resulting in tumorigenesis [23-25]. The fact that only a small proportion of individuals with HPV infection developed SCCHN suggests that genetic factors may potentially play a role in susceptibility to HPV-associated SCCHN. Our previous studies also have shown that genetic variants of genes involved in the p53 pathway, such as p53, p73 and MDM2, can modify the association of SCCHN risk with HPV seropositivity [26-28]. However, there have been no published population studies investigating the association between functional polymorphisms in the *NOXA* or *MCL1* promoter and the risk of HPV-associated SCCHN.

Cumulating evidence indicates that HPV E6/E7 may regulate the expression of *NOXA* and *MCL1* in different cancer cells. Inhibition of either E6 or E6-associated protein can trigger a slight increase of NOXA levels, despite inducing a prominent accumulation of p53, in HeLa human cervix cancer cell [29]. More recently, Cheng et al. [30] proved that, through the PI3K pathway, MCL1 expression levels are up-regulated in HPV E6- and E7- expressing and HPV16-infected cancer cells; and consistently, MCL1 expression is significantly reduced by knockdown of HPV E6 or E7 expression [31]. In addition, the promoter regions of both *NOXA* and *MCL1* contain an array of confirmed or putative transcription factor binding sites, such as p73, E2F1, and STAT response elements [7,32]. Since both *NOXA* and *MCL1* can be transcription-regulated, we hypothesized that HPV infection may deregulate NOXA-MCL1 axis-mediated apoptosis and subsequently affect the risk of SCCHN.

To test this hypothesis, we genotyped the putatively functional polymorphisms in the *NOXA* and *MCL1* promoters, and aimed to determine whether these genetic variants can, independently or jointly with HPV16 seropositivity, influence the risk of SCCHN, especially for those arising at the oropharyngeal site.

## Methods

### Patients and control samples

Details of this SCCHN case–control study population were previously described elsewhere [27]. Briefly, all histopathologically confirmed incident SCCHN patients were recruited consecutively as part of an ongoing molecular epidemiology study of SCCHN at the Head and Neck Surgery Clinic at The University of Texas M.D. Anderson Cancer Center between May 1996 and May 2002. The response rate of eligible patients who signed an informed agreement for participating in the study was approximately 95%. Excluded patients included those with second primary tumors; primary tumors of the sinonasal tract and nasopharynx; primary tumors outside the upper aerodigestive tract; cervical metastases of unknown origin; and histopathologic diagnoses of tumors other than squamous cell carcinoma, as well as patients with known immune symptoms, who had received recent blood transfusions within the last 6 months, or who were receiving immunosuppressive therapy. Finally, 380 non-Hispanic white SCCHN patients in whom HPV serology had been performed were included in this study.

Controls were recruited from a pool of cancer-free subjects from the hospitals in Houston metropolitan area. In this cancer-free control pool, a short questionnaire was used to determine each individual’s willingness to participate in the study before he or she was interviewed and asked to provide demographic and epidemiologic information including age, sex, ethnicity, smoking history, and alcohol consumption. Exclusion criteria for the control group included having had cancer previously, having known immune symptoms, received blood transfusions within the last 6 months, or were currently receiving immunosuppressive therapy. Approximately 78% of eligible subjects responded to the survey. As a result, 335 cancer-free control individuals were selected from the pool of potential controls by frequency matching on age (±5 years), gender, ethnicity, and smoking and alcohol drinking status. These variables were further adjusted in later multivariable logistic regression analyses to control for any residual confounding.

The “smoker” and “drinker” are defined in consistent with CDC report [33] and our previously published studies [26-28]. Briefly, “ever smokers” were those who had smoked more than 100 cigarettes in their lifetime, and the rest were “never smokers”; “ever drinkers” were those who drank alcoholic beverages at least once per week lasting for more than one year, and others were “never drinkers”. After a written informed consent was given, each individual provided a one-time 30-ml blood sample collected in heparinized tubes. The research protocol was approved by the institutional review board of M.D. Anderson Cancer Center.

### Selection and genotyping of candidate SNPs

The NCBI dbSNP database (http://www.ncbi.nlm.nih.gov/projects/SNP) build 131 and bioinformatics tool (http://manticore.niehs.nih.gov/snpfunc) were used to identify potentially functional SNPs in *NOXA* and *MCL1* promoter regions. We first scanned for all reported common SNPs (minor allele frequency, MAF > 0.05 in CEU population) that are predicted to affect the function of binding to transcription factors and located in the coding region or in the 5’-untranslated region known as the transcriptional regulatory region in the upstream of the start codon (from −2000 to +1), as well as for SNPs located in 3’-UTR in the downstream of coding region (+1 kb downstream) for potential miRNA binding sites; and then the linkage disequilibrium (LD) between pro-target SNPs was assessed by using the public HapMap SNP database (http://www.hapmap.org). As a result, of the 64 SNPs reported in *NOXA* and 91 SNPs reported in *MCL1*, there were no common non-synonymous SNPs located in the coding regions and no common SNPs located in the 3’-UTR that may potentially alter the miRNA-binding profile; finally, there were two SNPs in the *NOXA* promoter region (rs9957673, rs4558496) and two SNPs in the *MCL1* promoter region (rs9803935, rs4558496) predicted to be potentially functional and selected for genotyping. Genomic DNA obtained from the buffy coat of whole blood samples was extracted by the Qiagen DNA blood mini kit (Valencia, CA). Genotyping of *NOXA* and *MCL1* polymorphisms was performed by using the TaqMan assay, and the pre-designed probes were purchased from Applied Biosystems (ABI, Foster City, CA, USA). The pre-designed SNP genotyping assay IDs are C_30385519_20 for rs9957673, C_27902210_10 for rs4558496, C_1545306_10 for rs9803935, and C_27471449_10 for rs3738485. PCRs were run in a final volume of 5 μl per reaction, containing 10 ng genomic DNA, TaqMan Universal Master Mix, 80 × SNP Genotyping Assay Mix, and DNAse-free water. Four blank and positive controls and three random repeated samples were included in each plate for the genotyping assay quality control. The samples were genotyped by using the ABI 7900HT Sequence Detection System and Prism SDS 2.3 software.

### HPV16 serologic testing

In current study, a standard enzyme-linked immunoadsorption assay, as previously described, was used to identify the HPV16 L1 seropositivity [34]. The plasma HPV16 L1 capsid protein antibody was tested by using HPV16 L1 virus-like particles generated from recombinant baculovirus-infected insect cells. The HPV16 L1 seropositivity cutoff level was determined by using a standard pooled serum known to be at the cutoff point for HPV16 L1 seropositivity in a previous study [34]. Samples within 15% of the cutoff point were tested two more times, only those defined as positive in all three tests were considered positive. To avoid the heparin’s interfering for binding, plasma samples were treated with heparinase I before testing. No discernible reaction-difference was detected between the serum samples and the heparinized plasma samples obtained from three individuals. For quality control, 10% of the samples were randomly chosen for retesting and 100% concordance was observed in the repeat tests.

### Statistical analysis

The differences in distributions of selected variables, HPV16 serological status and *NOXA* and *MCL1* genotypes between cases and controls were examined by using the χ^2^ test. Odds ratios (OR) and their 95% confidence intervals (CI) were calculated to evaluate the association of *NOXA* and *MCL1* genotypes and HPV16 seropositivity with the risk of SCCHN in both univariate and multivariable logistic regression models. The joint effects of examined genotypes and HPV16 seropositivity on risk of SCCHN were evaluated, which were further assessed in subgroups stratified by tumor sites, age, smoking and drinking status. The “linkage disequilibrium” (LD) procedure in the HelixTree v7.3.0 (Golden Helix Inc., Bozeman, MT, USA) was used to estimate the LD by means of LD coefficients (D’) and the *r*^*2*^*.* All of the statistical analyses were performed by using Statistical Analysis System software (Version 9.1; SAS Institute, Cary, NC), and two-sided *P* value < 0.05 was considered statistically significant.

## Results

### Demographic and risk factors for study subjects

We originally recruited 380 eligible cases and 335 controls of non-Hispanic whites in this study, of these subjects, eight cases and twenty controls failed in the genotyping assays. Therefore, 372 cases and 315 controls were included in the final genotyping analysis. The demographic characteristics and distribution of known SCCHN risk factors of the study population are summarized in Table 1. The cases and controls appeared to be adequately frequency-matched for sex, age, smoking status, and alcohol use. However, we found that cases were much more likely than controls to be HPV16 seropositive (*P* < 0.0001). HPV16 seropositivity was significantly associated with increased risk of oropharyngeal cancer (adjusted OR = 5.7; 95% CI, 3.6-8.9) but not SCCHN at non-oropharyngeal sites (adjusted OR = 0.7; 95% CI, 0.4-1.3).

**Table 1:** Frequency distribution of demographic and risk factors in SCCHN cases and controls

**Characteristics**	**Patients (n = 380)**		**Controls^a^ (n = 335)**		***P* value^b^**
	**No.**	**%**	**No.**	**%**	
Age (years)					0.526
<40	32	8.4	27	8.1	
41–55	142	37.4	109	32.5	
56–70	159	41.8	157	46.9	
>70	47	12.4	42	12.5	
Sex					0.100
Male	285	75.0	269	80.3	
Female	95	25.0	66	19.7	
Ethnicity					
Non-Hispanic white	380	100.0	335	100.0	
Tobacco smoking					0.588
Ever	278	73.2	239	71.3	
Never	102	26.8	96	28.7	
Alcohol drinking					
Ever	296	77.9	240	71.6	0.054
Never	84	22.1	95	28.4	
Tumor site					
Oropharyngeal	187	49.2			
Non-Oropharyngeal	193	50.8			

^a^ The controls were frequency matched to the patients on the factors shown in this table.

^b^ Two-sided *χ*^2^ test.

### Joint effects of gene variants and HPV16 seropositivity on the risk of SCCHN

The distributions of *NOXA* and *MCL1* genotypes and their associations with risk of SCCHN are shown in Table 2. The frequencies of all tested SNPs among controls were in agreement with Hardy-Weinberg equilibrium (*P =* 0.069 for rs9957673, *P =* 0.139 for rs4558496, *P =* 0.898 for rs9803935, and *P =* 0.871 for rs3738485). Because the LD evaluation for the SNPs in *NOXA* or *MCL1* is unavailable in the HpaMap database, we calculated the LD values for the SNPs studied. The *NOXA* rs9957673 and *NOXA* rs4558496 were in low linkage (D’ = 0.901, *r*^2^ = 0.04), while the *MCL1* rs9803935 and *MCL1* rs3738485 were in high linkage (D’ = 0.98, *r*^2^ = 0.95). There was no significant association observed between the putatively functional SNPs and the risk of overall or subtypes of the SCCHN (oropharyngeal or non-oropharyngeal cancers). However, we found that the variants of both *NOXA* and *MCL1* genes modified the association between the HPV16 seropositivity and the risk of SCCHN.

**Table 2:** **Associations between*NOXA*and*MCL1*genotypes and SCCHN risk**

**Gene Variant^a^**	**Control**	**Overall (n = 372)**		**Oropharynx (n = 184)**		**Non-oropharynx (n = 188)**	
	**n = 315 (%)**	**Patients (%)**	**OR^b^ (95%CI)**	**Patients (%)**	**OR^b^ (95%CI)**	**Patients (%)**	**OR^b^ (95%CI)**
*NOXA* rs9957673							
CC	216 (68.6)	246 (66.1)	1.0	118 (64.1)	1.0	128 (68.1)	1.0
CT	95 (30.2)	115 (30.9)	1.0 (0.7-1.4)	60 (32.6)	1.1 (0.7-1.7)	55 (29.3)	1.1 (0.9-1.6)
TT	4 (1.3)	11 (3.0)	2.3 (0.7-7.4)	6 (3.3)	1.9 (0.4-8.1)	5 (2.7)	2.0 (0.5-8.3)
			*P*_trend_ =0.300		*P*_trend_ = 0.180		*P*_trend_ = 0.689
CT + TT	99 (31.4)	126 (33.9)	1.1 (0.8-1.5)	66 (35.9)	1.1 (0.7-1.7)	60 (31.9)	1.1 (0.7-1.6)
*NOXA* rs4558496							
TT	216 (68.6)	253 (69.1)	1.0	121 (65.8)	1.0	136 (72.3)	1.0
TG	85 (27.5)	107 (29.3)	1.0 (0.7-1.5)	61 (33.2)	1.23 (0.8-1.9)	48 (25.5)	0.9 (0.6-1.3)
GG	14 (4.3)	6 (1.6)	0.4 (0.2-1.1)	2 (1.1)	0.34 (0.1-1.6)	4 (2.1)	0.4 (0.1-1.4)
			*P*_trend_ =0.411		*P*_trend_ = 0.913		*P*_trend_ = 0.224
TG + GG	99 (31.4)	115 (30.9)	1.0 (0.7-1.3)	63 (34.3)	1.1 (0.7-1.7)	52 (27.7)	0.8 (0.5-1.2)
*MCL1* rs9803935							
GG	95 (30.2)	127 (34.1)	1.0	65 (35.3)	1.0	62 (33.0)	1.0
GT	157 (59.8)	169 (45.4)	0.8 (0.6-1.2)	77 (41.9)	0.8 (0.5-1.3)	92 (48.9)	0.9 (0.6-1.4)
TT	63 (20.0)	76 (20.4)	0.9 (0.6-1.5)	42 (22.8)	1.1 (0.6-1.8)	34 (18.1)	0.9 (0.6-1.6)
			*P*_trend_ =0.516		*P*_trend_ = 0.726		*P*_trend_ = 0.463
GG + GT	252 (80.0)	296 (79.6)	1.0	142 (77.2)	1.0	154 (81.9)	1.0
TT	63 (20.0)	76 (20.4)	1.0 (0.7-1.5)	42 (22.8)	1.2 (0.7-1.9)	34 (18.1)	1.0 (0.6-1.6)
*MCL1* rs3738485							
GG	93 (29.5)	128 (34.4)	1.0	65 (35.3)	1.0	63 (33.5)	1.0
GC	155 (49.2)	168 (45.2)	0.8 (0.6-1.2)	77 (41.9)	0.8 (0.5-1.3)	91 (47.4)	0.9 (0.6-1.3)
CC	67 (21.3)	76 (20.4)	0.9 (0.6-1.3)	42 (22.8)	1.0 (0.6-1.7)	34 (18.1)	0.9 (0.5-1.5)
			*P*_trend_ =0.298		*P*_trend_ = 0.528		*P*_trend_ = 0.271
GG + GC	248 (78.7)	296 (79.6)	1.0	142 (77.2)	1.0	154 (81.9)	1.0
CC	67 (21.3)	76 (20.4)	1.0 (0.7-1.4)	42 (22.8)	1.1 (0.7-1.8)	34 (18.1)	0.9 (0.6-1.5)

^a^ The observed genotype frequencies among the controls were in agreement with Hardy-Weinberg equilibrium (*P*=0.069 for rs9957673, *P*=0.139 for rs4558496, *P*=0.898 for rs9803935, *P*=0.871 for rs3738485).

^b^ ORs were adjusted for age, sex, smoking, drinking, and HPV16 serology.

As summarized in Table 3, we estimated the joint effects of the HPV16 seropositivity and genotypes of *NOXA* and *MCL1* on SCCHN risk. By using the group with the *NOXA* rs9957673 CC genotype and HPV16 seronegativity as reference, those with the CT/TT genotypes and HPV16 seronegativity did not show association with increased SCCHN risk (OR = 1.0; 95% CI,0.7-1.4); however, those with the CC genotype and HPV16 seropositivity had a 2.4-fold increased SCCHN risk (95% CI, 1.4-4.0), and those with the CT/TT genotypes and HPV16 seropositivity had a 3.6-fold increased SCCHN risk (95% CI,1.9-6.8). Similarly, compared with those with the *NOXA* rs4558496 TT genotype and HPV16 seronegativity, those with the TT genotype and HPV16 seropositivity had an OR of 2.7 (95% CI, 1.4-5.4), and those with both TG/GG genotypes and HPV16 seropositivity had an OR of 2.8 (95% CI, 1.4-5.5). For the two linked SNPs in *MCL1* promoter, when subjects with the *MCL1* rs9803935 GG/GT genotypes and HPV16 seronegativity as the reference group, those with the TT genotype and HPV16 seronegativity had an OR of 0.8 (95% CI, 0.5-1.2), those with the GG/GT genotypes and HPV16 seropositivity had an OR of 2.1 (95% CI, 1.3-3.3), and those with the TT genotype and HPV16 seropositivity had an OR of 8.1 (95% CI, 2.8-23.7). The similar results were also observed for *MCL1* rs3738485 polymorphism (Table 3).

**Table 3:** **Joint effects between HPV16 seropositivity and*NOXA and MCL1*genotypes on risk of SCCHN**

**HPV16 Serology**	**Gene Variant**	**Control**	**Overall (n = 372)**		**Oropharynx (n = 184)**		**non-oropharynx (n = 188)**	
		**n = 315 (%)**	**Patients (%)**	**OR^a^ (95%CI)**	**Patients (%)**	**OR^a^ (95%CI)**	**Patients (%)**	**OR^a^ (95%CI)**
	*NOXA* rs9957673							
-	CC (Ref.)	189 (60.0)	187 (50.3)	1.0	68 (37.4)	1.0	119 (63.3)	1.0
-	CT + TT	85 (27.0)	83 (22.3)	1.0 (0.7-1.4)	31 (17.0)	1.0 (0.6-1.7)	52 (27.7)	1.0 (0.7-1.5)
+	CC	27 (8.6)	59 (15.9)	2.4 (1.4-4.0)	50 (27.2)	4.8 (2.8-8.3)	9 (4.8)	0.5 (0.2-1.2)
+	CT + TT	14 (4.4)	43 (11.6)	3.6 (1.9-6.8)	35 (19.0)	7.1 (3.6-14.3)	8 (4.3)	1.1 (0.4-2.8)
				*P*_trend_ =0.017		*P*_trend_ < 0.001		*P*_trend_ = 0.744
	*NOXA* rs4558496							
-	TT (Ref.)	188 (59.7)	188 (50.8)	1.0	66 (36.3)	1.0	123 (65.4)	1.0
-	TG + GG	86 (27.3)	86 (21.8)	0.9 (0.6-1.4)	33 (17.9)	1.1 (0.7-1.8)	48 (25.5)	0.8 (0.5-1.3)
+	TT	28 (8.9)	68 (18.3)	2.7 (1.7-4.5)	55 (29.9)	5.6 (3.2-9.6)	13 (6.9)	0.8 (0.4-1.7)
+	TG + GG	13 (4.1)	34 (9.1)	2.8 (1.4-5.5)	30 (16.3)	6.1 (3.0-12.6)	4 (2.1)	0.4 (0.1-1.4)
				*P*_trend_ =0.086		*P*_trend_ < 0.001		*P*_trend_ = 0.197
	*MCL1* rs9803935							
-	GG + GT (Ref.)	215 (68.2)	224 (60.2)	1.0	81 (44.0)	1.0	143 (76.1)	1.0
-	TT	59 (18.7)	46 (12.4)	0.8 (0.5-1.2)	18 (9.8)	0.8 (0.5-1.5)	28 (14.9)	0.8 (0.5-1.3)
+	GG + GT	37 (11.8)	72 (19.4)	2.1 (1.3-3.3)	61 (33.2)	4.3 (2.6-7.1)	11 (5.9)	0.5 (0.2-1.0)
+	TT	4 (1.3)	30 (8.1)	8.1 (2.8-23.7)	24 (13.0)	14.9 (5.0-44.6)	6 (3.2)	2.7 (0.7-9.8)
				*P*_trend_ =0.030		*P*_trend_ < 0.001		*P*_trend_ = 0.317
	*MCL1* rs3738485							
-	GG + GC (Ref.)	212 (67.3)	223 (60.0)	1.0	81 (44.0)	1.0	142 (75.5)	1.0
-	CC	62 (19.7)	47 (12.6)	0.8 (0.5-1.2)	18 (9.8)	0.8 (0.4-1.4)	29 (15.4)	0.8 (0.5-1.3)
+	GG + GC	36 (11.4)	73 (19.6)	2.2 (1.4-3.4)	61 (33.2)	4.4 (2.7-7.2)	12 (6.4)	0.5 (0.3-1.1)
+	CC	5 (1.6)	29 (7.8)	6.3 (2.4-16.7)	24 (13.0)	11.7 (4.3-32.0)	5 (2.7)	1.9 (0.5-6.8)
				*P*_trend_ =0.071		*P*_trend_ < 0.001		*P*_trend_ = 0.189

^a^ ORs were adjusted for age, sex, and tobacco smoking and alcohol drinking.

We further analyzed the joint effects between the SNPs and HPV16 seropositivity on the risks of both oropharyngeal and non-oropharyngeal cancers. We found that effect modification of the *NOXA* and *MCL1* SNPs on risk of HPV-associated cancers was evident for oropharyngeal but not for SCCHN at non-oropharyngeal sites (Table 3).

### Stratification of the joint effects of NOXA and MCL1 variants and HPV16 seropositivity on oropharyngeal cancer risk

Since the modification effect of *NOXA* and *MCL1* variants on SCCHN risk associated with HPV16 seropositivity was particularly evident for oropharyngeal cancers, we further evaluated the effect modification of the variants on risk of oropharygeal cancer associated with HPV16 seropositivity stratified by smoking/drinking status and age (Table 4, 5, 6).

**Table 4:** **Joint effect between HPV16 seropositivity and*NOXA*and*MCL1*genotypes on risk of oropharyngeal cancer stratified by smoking status**

**HPV16 Serology**	**Genotypes**	**Never smokers**		**Ever smokers**		**Adjusted OR (95% CI) ^a^**	
		**Patients (n = 61)**	**Controls (n = 90)**	**Patients (n = 123)**	**Controls (n = 225)**	**Never smokers**	**Ever smokers**
	*NOXA* rs9957673						
-	CC (Ref.)	19	52	49	137	1.0	1.0
-	CT + TT	7	30	24	55	0.7 (0.2-1.8)	1.2 (0.7-2.2)
+	CC	18	7	32	20	8.3 (2.8-24.9)	4.3 (2.4-8.4)
+	CT + TT	17	1	18	13	50.6 (6.1-422.0)	4.1 (1.9-9.2)
						*P*_trend_ = 0.001	*P*_trend_ = 0.004
	*NOXA* rs4558496						
**-**	TT (Ref.)	19	57	47	131	1.0	1.0
**-**	TG + GG	7	25	26	61	0.7 (0.2-2.0)	1.2 (0.7-2.1)
+	TT	26	7	29	21	13.4 (4.7-37.8)	4.0 (2.0-7.7)
+	TG + GG	9	1	21	12	21.9 (2.5-192.6)	4.7 (2.1-10.5)
						*P*_trend_ = 0.008	*P*_trend_ = 0.002
	*MCL1* rs3738485						
-	GG + GT (Ref.)	20	66	61	149	1.0	1.0
-	TT	6	16	12	43	1.4 (0.5-4.3)	0.7 (0.3-1.4)
+	GG + GT	27	7	34	30	15.4 (5.5-43.6)	2.8 (1.6-5.1)
+	TT	8	1	16	3	28.0 (3.2-246.6)	12.1 (3.4-43.3)
						*P*_trend_ < 0.001	*P*_trend_ = 0.003
	*MCL1* rs3738485						
**-**	GG + GC (Ref.)	20	65	61	147	1.0	1.0
**-**	CC	6	17	12	45	1.3 (0.4-4.1)	0.6 (0.3-1.3)
+	GG + GC	27	7	34	29	15.3 (5.4-43.5)	2.9 (1.6-5.2)
+	CC	8	1	16	4	27.7 (3.1-244.7)	8.8 (2.8-27.8)
						*P*_trend_ < 0.001	*P*_trend_ = 0.008

^a^ ORs were adjusted for age, sex, and alcohol drinking.

**Table 5:** **Joint effect between HPV16 seropositivity and*NOXA*and*MCL1*genotypes on risk of oropharyngeal cancer stratified by drinking status**

**HPV16 Serology**	**Genotypes**	**Never drinkers**		**Ever drinkers**		**Adjusted OR (95% CI) ^a^**	
		**Patients (n = 36)**	**Controls (n = 90)**	**Patients (n = 148)**	**Controls (n = 225)**	**Never drinkers**	**Ever drinkers**
	*NOXA* rs9957673						
-	CC (Ref.)	14	57	54	132	1.0	1.0
-	CT + TT	3	24	28	61	0.5 (0.1-1.8)	1.2 (0.7-2.0)
+	CC	9	5	41	22	8.1 (2.2-29.5)	4.3 (2.3-8.0)
+	CT + TT	10	4	25	10	12.7 (3.2-50.0)	5.9 (2.6-13.3)
						*P*_trend_ = 0.015	*P*_trend_ = 0.001
	*NOXA* rs4558496						
**-**	TT (Ref.)	9	56	57	132	1.0	1.0
**-**	TG + GG	8	25	25	61	1.8 (0.6-5.4)	1.0 (0.6-1.7)
+	TT	16	7	39	21	15.4 (4.8-49.2)	4.1 (2.2-7.7)
+	TG + GG	3	2	27	11	12.9 (1.7-99.5)	5.4 (2.5-11.6)
						*P*_trend_ = 0.019	*P*_trend_ = 0.001
	*MCL1* rs9803935						
-	GG + GT (Ref.)	12	65	69	150	1.0	1.0
-	TT	5	16	13	43	1.8 (0.5-5.8)	0.7 (0.3-1.4)
+	GG + GT	16	9	45	28	11.7 (3.9-34.7)	3.3 (1.9-5.8)
+	TT	3	NA	21	4	NA	10.8 (3.5-33.0)
						*P*_trend_ = 0.002	*P*_trend_ = 0.001
	*MCL1* rs3738485						
**-**	GG + GC (Ref.)	12	63	69	149	1.0	1.0
**-**	CC	5	18	13	44	1.6 (0.5-5.1)	0.7 (0.3-1.3)
+	GG + GC	16	9	45	27	11.4 (3.8-34.1)	3.4 (1.9-6.0)
+	CC	3	NA	21	5	NA	8.6 (3.1-23.9)
						*P*_trend_ = 0.760	*P*_trend_ = 0.001

^a^ ORs were adjusted for age, sex, and tobacco smoking.

**Table 6:** **Joint effect between HPV16 seropositivity and*NOXA*and*MCL1*genotypes on risk of oropharyngeal cancer stratified by age**

**HPV16 Serology**	**Genotypes**	**Age ≤ 58**		**Age > 58**		**Adjusted OR (95% CI) ^a^**	
		**Patients (n = 116)**	**Controls (n = 145)**	**Patients (n = 68)**	**Controls (n = 170)**	**Age ≤ 58**	**Age > 58**
	*NOXA* rs9957673						
-	CC (Ref.)	39	89	29	100	1.0	1.0
-	CT + TT	15	38	16	47	0.9 (0.5-2.0)	1.2 (0.6-2.5)
+	CC	36	14	14	13	6.4 (3.0-13.6)	4.0 (1.7-9.8)
+	CT + TT	26	4	9	10	14.9 (4.7-46.6)	3.6 (1.3-10.0)
						*P*_trend_ < 0.001	*P*_trend_ = 0.077
	*NOXA* rs4558496						
**-**	TT (Ref.)	36	90	30	98	1.0	1.0
**-**	TG + GG	18	37	15	49	1.2 (0.6-2.4)	1.0 (0.5-2.1)
+	TT	38	11	17	17	9.5 (4.2-21.2)	3.5 (1.6-7.9)
+	TG + GG	24	7	6	6	8.2 (3.2-21.3)	3.9 (1.1-13.8)
						*P*_trend_ < 0.001	*P*_trend_ = 0.222
	*MCL1* rs9803935						
-	GG + GT (Ref.)	44	102	37	113	1.0	1.0
-	TT	10	25	8	34	0.9 (0.4-2.0)	0.8 (0.3-1.8)
+	GG + GT	42	17	19	20	5.9 (3.0 -11.8)	3.3 (1.6-8.0)
+	TT	20	1	4	3	47.2 (6.1 -368.2)	3.9 (0.8-19.0)
						*P*_trend_ < 0.001	*P*_trend_ = 0.338
	*MCL1* rs3738485						
**-**	GG + GC (Ref.)	44	100	37	112	1.0	1.0
**-**	CC	10	27	8	35	0.8 (0.4-1.9)	0.7 (0.3-1.7)
+	GG + GC	42	16	19	20	6.2 (3.1-12.5)	3.3 (1.5-7.0)
+	CC	20	2	4	3	22.7 (5.0-103.5)	3.8 (0.8-18.7)
						*P*_trend_ < 0.001	*P*_trend_ = 0.270

^a^ ORs were adjusted for age, sex, tobacco smoking and alcohol drinking.

For each polymorphism, the modifying effects on the risk of oropharyngeal cancer associated with HPV16 seropositivity were much stronger in never smokers than in ever smokers (Table 4) and in never drinkers than in ever drinkers (Table 5). Additionally, in the age-stratified analysis (stratified by the mean age of controls), the modifying effects of *NOXA* and *MCL1* variants on risk of oropharyngeal cancer associated with HPV16 seropositivity was more pronounced among younger subjects (aged ≤ 58 year) than among older subjects (aged > 58 year) (Table 6).

## Discussion

In this study, we did not observe significant main effects of the SNPs in the promoter regions of *NOXA* and *MCL1* genes on the risk of SCCHN. However, all tested polymorphisms showed effect modification on risk of HPV-associated SCCHN, particularly for oropharyngeal cancer patients who were never smokers, never drinkers, and younger subjects (aged ≤ 58 year). Although these data suggest a potential joint effect between HPV16 infection and *NOXA* or *MCL1* polymorphisms, larger studies are still needed to validate our findings.

The deregulation of apoptosis is known as an important cause of neoplasia. In the intrinsic apoptotic pathway, apoptosis is usually mediated by cooperation among proapoptotic members (such as NOXA) and antiapoptotic members (such as MCL1) of the Bcl-2 family proteins. Differing from other BH3-only members, such as PUMA, NOXA usually executes its proapoptotic function by exclusively binding to MCL1, A1 or Bcl-_XL_, but with higher priority and affinity to MCL1 [2,7,35]. Studies from multiple cancer cells have shown that the NOXA inhibition or MCL1 overexpression can dramatically reduce apoptosis and consequently promote the tumor viability [36-38], which suggests a possible effect of NOXA-MCL1 axis on susceptibility to cancers. In consistence with this scenario, we found in current study that, to some extent, the risk of HPV16-associated SCCHN, particular oropharyngeal cancer, can be modified by genetic variants of *NOXA* and *MCL1*.

The decrease of SCCHN incidence in recent years accompanied by decline of tobacco use has not been observed for all head and neck cancer sites. However, the results from the America and European cohort studies suggested that the oropharyngeal cancer incidence (especially in younger white populations) has steadily increased and shown to be associated with the involvement of HPVs, particularly the high risk type 16 [17,19,39-41]. The HPV oncoproteins E6 and E7 may play decisive roles in HPV16-associated SCCHN by E6-mediated p53 degradation or E7-mediated pRb-E2F degradation and disruption of the p53-related or pRb-related pathway [23,24].

Interestingly, *NOXA* and *MCL1* are targets of p53 and E2F, and p53 can affect the expression of its instant downstream target *NOXA* by directly binding to the promoter response element. In addition, other common transcription factors, such as E2F and p73, may also transcriptively regulate the expression of *NOXA* as well as *MCL1*, through respective response elements in the promoter regions of *NOXA* or *MCL1*[7,32], thereby altering susceptibility to SCCHN. It is therefore mechanically conceivable and biologically plausible that HPV16 infection and the functional SNPs in the promoters of *NOXA* and *MCL1* may jointly affect apoptosis induction, accordingly altering susceptibility to SCCHN. In agreement with that, we did observe a pronounced modification effect of these genetic variants on the risk of SCCHN, especially oropharyngeal cancer, associated with HPV16 seropositivity.

Some other studies provided evidence for such kind of joint effects. In a very recent study, by blocking HPV E6-mediated p53 degradation to activate the transcription of *NOXA*, RITA (a small-molecule reactivation of p53 and induction of tumor cell apoptosis) induced apoptosis in multiple cancer cells containing HPV16 and substantially suppressed the growth of cervical carcinoma xenografts in vivo [29]. Not only the E6 oncoprotein, but the E7 oncoprotein can also interact with *NOXA*. By disrupting pRb-E2F complexes, the expression of HPV16 E7 protein up-regulates the *NOXA* expression, whereas, the binding of E2F to the *NOXA* promoter results in a significant reduction of *NOXA*-mediated apoptosis [42], suggesting that HPV E7 may indeed deregulate the *NOXA* expression. In addition, HPV E6- or E7-transfection can significantly up-regulate the expression of *MCL1,* therefore promoting lung tumor cell progression through PI3K pathway [30,31]. These data suggest a possibility that, by deregulating apoptosis and inferring tumor growth, HPV16 infection may alter the progress of NOXA-MCL1 axis-mediated carcinogenesis through both p53- dependent and independent mechanisms. Indeed, we observed in current study that the risk associated with the joint effects between HPV16 seropositivity and variants of *NOXA* and *MCL1* dramatically increased for SCCHN at the oropharyngeal (HPV-related) site but not at non-oropharyngeal (HPV-unrelated) sites. Although our study implied significant trends of effect modification, we did not have adequate power to detect the interaction.

In consistence with our previously published studies in which HPV16 and genetic variants of p53 pathway genes, such as *p53* or *MDM2*, may have interactive effects on risk of oropharyngeal cancer, particularly in never smokers and never drinkers [27,28], we also found in current study more pronounced modification effects of *NOXA* and *MCL1* variants on the risk associated with HPV16 seropositivity for oropharyngeal cancer, particularly in never smokers and never drinkers. In addition, when the subjects were dichotomized by the mean age of the controls, we observed more apparent joint effects in younger, than older subjects. Though the mechanism is unclear, many researchers recently documented an apparent increase of incidence of HPV-related oropharyngeal cancer, particularly in young adults, perhaps due to distinct changes in sexual behaviors [43,44]. To the best of our knowledge, the current study is the first to evaluate the association between polymorphisms in the promoter region of *NOXA* and *MCL1* and risk of SCCHN, as well as the joint effects between the genetic variants and HPV16 infection in the etiology of SCCHN.

However, there are several limitations in our present study. Firstly, although our results suggest a potential effect on SCCHN risk associated with HPV16, the underlying mechanism of *NOXA* and *MCL1* in the development of SCCHN is still unclear. Thus future studies are needed to focus on how these polymorphisms affect functional changes of the two genes. Secondly, the HPV16 serology assay used in our study could test the pre-exposure status but could not define the accurate infected organs or determine if the viral exposure took place before or after tumor development. Therefore, with this uncertainty applied to both the cases and controls, possible false-negative HPV16 cases might result in misclassification of the HPV16 status. However, the use of serology assay allowed for the inclusion of a cancer-free control group. Additionally, the relative small sample size could not provide enough statistical power for further stratified analysis or interaction analysis; thus we cannot exclude the possibility that our results could be by chance. Finally, we cannot deduce similar conclusions with certainty to other ethnic populations, because our study included only non-Hispanic white participants.

## Conclusions

We found that putatively functional polymorphisms of *NOXA* and *MCL1* may modify the risk of SCCHN associated with HPV seropositive, especially the risk of oropharyngeal cancer among never smokers, never drinkers and younger individuals. Regardless of the mechanism, our results implied that a potential joint effect among *NOXA*, *MCL1* variants and HPV16 seropositivity may contribute the SCCHN risk. The identification of population subgroups at higher risk provide evidence that HPV-targeting treatment may help benefit SCCHN. However, well-designed studies with larger sample sizes in different ethnic populations are needed to validate our findings.

## Pre-publication history

The pre-publication history for this paper can be accessed here:

http://www.biomedcentral.com/1471-2407/12/159/prepub
